# Treatment of the benign lytic lesions of the proximal femur with synthetic bone graft

**DOI:** 10.1186/s13018-018-0982-z

**Published:** 2018-10-29

**Authors:** Karem M. Zekry, Norio Yamamoto, Katsuhiro Hayashi, Akihiko Takeuchi, Ali Zein A. A. Alkhooly, Ahmed Saleh Abd-Elfattah, Ezzat H. Fouly, Adel Refaat Ahmed, Hiroyuki Tsuchiya

**Affiliations:** 1 Department of Orthopaedic Surgery, Graduate School of Medical Science, Kanazawa University, Kanazawa, Japan; 2 Department of Orthopaedic Surgery, Faculty of Medicine, Minia University, El-Minya, Egypt; 3 Department of Orthopaedic Surgery, Faculty of Medicine, Alexandria University, Alexandria, Egypt

**Keywords:** Benign lytic lesions, Proximal femur, Synthetic bone graft

## Abstract

**Background:**

Benign bone tumors and tumor-like conditions are commonly located in the proximal femur. The main indications for surgical treatment are lesions with impending or actual pathological fractures, or with aggressive or recurrent lesions. However, patients complaining of persistent pain, limping, or abnormal gait patterns are also considered for surgical treatment. In this study, we describe the outcomes of the surgical treatment of benign lytic lesions of the proximal femur by curettage followed by implantation of synthetic bone graft.

**Methods:**

This retrospective study included 27 patients (22 females and 5 males) with benign lytic lesions of the proximal femur. The average age was 25.5 years (6–65 years), and the mean follow-up period was 54.5 months (9–145 months). The histopathological diagnoses were fibrous dysplasia (8 patients), simple bone cyst (8 patients), chondroblastoma (7 patients), giant cell tumor (3 patients), and eosinophilic granuloma (1 patient). These lesions were managed with curettage followed by implantation of the bone defects with alpha tricalcium phosphate in 14 patients, beta tricalcium phosphate granules in 11 patients, hydroxyapatite granules in 1 patient, and combined beta tricalcium phosphate and hydroxyapatite granules in 1 patient. Internal fixation was performed in three patients.

**Results:**

The mean operative time was 143 min (80–245 min). Patients had regained normal unrestricted activity without pain at the operation site. Patients treated with beta tricalcium phosphate achieved radiographic consolidation of the bone defects within 1 year after the surgery, and those treated with alpha tricalcium phosphate or hydroxyapatite experienced no progression nor recurrence of the lesions. Local tumor recurrence was observed in one patient with giant cell tumor 5 years after the surgery. Post-operative pathological fracture was occurred in one patient with a simple bone cyst of the subtrochanteric region 1 month after surgery. No post-operative infection was observed.

**Conclusion:**

We concluded that the treatment of benign lytic lesions of the proximal femur, either primary or recurrent, using synthetic bone graft is a safe and satisfactory method and the addition of internal fixation should be carefully planned.

## Background

Benign bone tumors and tumor-like conditions are commonly located in the proximal femur [1] with high risk of pathological fracture [2]. It has been reported that destruction of more than 54% of the bone cortex by tumor carries a high risk for pathological fracture [3].

Benign bone lesions as giant cell tumor (GCT), simple bone cyst (SBC), fibrous dysplasia, chondroblastoma, and aneurysmal bone cyst (ABC) are commonly seen in this region. Surgical treatment is usually indicated in lesions with impending or actual pathological fractures, or with aggressive or recurrent lesions. However, patients complaining of persistent pain, limping, or abnormal gait patterns are also considered for surgical treatment. Surgical treatment options for benign lytic lesions of the proximal femur include curettage, and bone grafting of the resulting defect with or without internal fixation. Most of these studies recommended either autogenous or allogenic bone graft. Local adjuvants may be used to control local recurrence of the aggressive lesions [1, 2, 4–8]. Some recent studies either in vivo or in vitro trials report the efficacy of synthetic bone substitute for the reconstruction of bone defects after curettage of benign lesions [9, 10].

This retrospective study was designed to describe the outcomes of the surgical treatment of benign lytic lesions of the proximal femur by curettage followed by implantation of synthetic bone graft.

## Methods

Between 1996 and 2015, 27 patients (22 females and 5 males) with benign lytic lesions of the proximal femur, underwent surgical treatment with curettage followed by implantation of synthetic bone graft (Table 1). The indications for surgical treatment of these lesions were impending pathological fracture, aggressive benign lesions such as GCT, persistent pain and limping, or recurrent or residual lesions after previous treatment other than curettage and implantation of synthetic bone graft.

**Table 1:** Characteristic details of patient

No.	Age and gender	Diagnosis	Synthetic substitute	Internal fixation	Follow-up (months)	Operative time (min)	Complication
1	18/M	Residual SBC	HA	No	76	100	–
2	31/F	FD	β-TCP -HA	No	145	135	–
3	41/M	SBC	β-TCP	No	9	80	–
4	11/M	FD	β-TCP	No	57	140	–
5	28/M	EG	β-TCP	No	14	140	–
6	21/M	SBC	β-TCP	No	52	95	–
7	65/M	FD	β-TCP	No	42	215	–
8	22/M	CB	β-TCP	No	10	150	–
9	18/F	GCT	β-TCP	No	98	245	Recurrence
10	32/M	CB	β-TCP	No	127	95	–
11	16/M	CB	α-TCP	No	27	150	–
12	28/M	CB	β-TCP	DHS	144	210	–
13	6/M	SBC	β-TCP	No	30	82	–
14	22/F	SBC	α-TCP	No	64	100	–
15	6/M	Recurrent SBC	α-TCP	No	81	92	–
16	21/M	CB	α-TCP	No	35	156	–
17	24/M	Recurrent GCT	α-TCP	Screw	77	212	–
18	32/M	GCT	α-TCP	No	91	220	–
19	20/M	CB	α-TCP	No	26	131	–
20	15/M	Recurrent CB	α-TCP	No	39	157	–
21	12/M	FD	α-TCP	No	54	174	–
22	37/F	FD	α-TCP	No	41	141	–
23	36/M	FD	α-TCP	No	26	148	–
24	27/F	FD	α-TCP	No	38	115	–
25	16/M	SBC	α-TCP	No	33	114	Fracture
26	46/M	FD	β-TCP	DHS	27	123	–
27	38/M	SBC	α-TCP	No	10	139	–

*α-TCP* alpha tricalcium phosphate, *β-TCP* beta tricalcium phosphate, *CB* chondroblastoma, *DHS* dynamic hip screw, *EG* eosinophilic granuloma, *F* female, *FD* fibrous dysplasia, *GCT* giant cell tumor, *HA* hydroxyapatite, *M* male, *SBC* simple bone cyst

The patients were followed-up for a mean period of 54.5 (9–145) months. The average age of the patients was 25.5 (6–65) years. The histopathological diagnoses were fibrous dysplasia (8 patients), simple bone cyst (SBC) (6 patients), chondroblastoma (6 patients), GCT (2 patients), eosinophilic granuloma (1 patient), residual SBC after previous treatment of continuous decompression with hydroxyapatite cannulated pins (1 patient), recurrent SBC after previous treatment of continuous decompression (1 patient), recurrent GCT after previous treatment with cementation (1 patient) and recurrent chondroblastoma after previous treatment with curettage in another hospital (1 patient).

These lesions were managed with curettage followed by implantation of the bone defects with alpha tricalcium phosphate (α-TCP) in 14 patients, beta tricalcium phosphate (β-TCP) granules in 11 patients, hydroxyapatite (HA) granules in 1 patient, and combined β-TCP and HA granules in 1 patient (α-TCP and HA granules are products of Hoya corporation. β-TCP granules are products of Plympus corporation). Internal fixation was performed in three lesions; two lesions were fixed with dynamic hip screws and one lesion with a cannulated screw. The internal fixation was indicated in large lesions with preoperative impending pathological fracture due to significant endosteal erosion of the cortex with marked thinning of the wall of the lesion.

A preoperative work-up in all patients included a history, clinical examination, radiography of the lesion, computed tomography (CT), magnetic resonance imaging (MRI) and routine laboratory tests, but in patients with aggressive lesions, CT of the chest and technetium-99m and thallium-201 bone scintigraphy may be indicated. Ethical approval for this study was granted from the Institutional Review Board of Kanazawa University, and written informed consent was obtained from all patients.

### Surgical procedure

The choice of surgical approach depended on the location of the lesion within the proximal femur. If the lesion involved the trochanteric or subtrochanteric region, the operation was performed through a lateral approach, but if the lesion involved only the neck or head of the femur, the operation was performed through an anterior approach.

After approaching the lesion, a cortical window was created either through the lateral femoral cortex or through the anterior cortex of the neck depending on location of the lesion and the chosen surgical approach. Through this cortical window, a biopsy was obtained for frozen section. If the pathological diagnosis corresponded with the preoperative imaging diagnosis, the definitive surgical operation was performed; otherwise, the definitive surgical procedure was delayed until the diagnosis was confirmed.

**Fig. 1:**
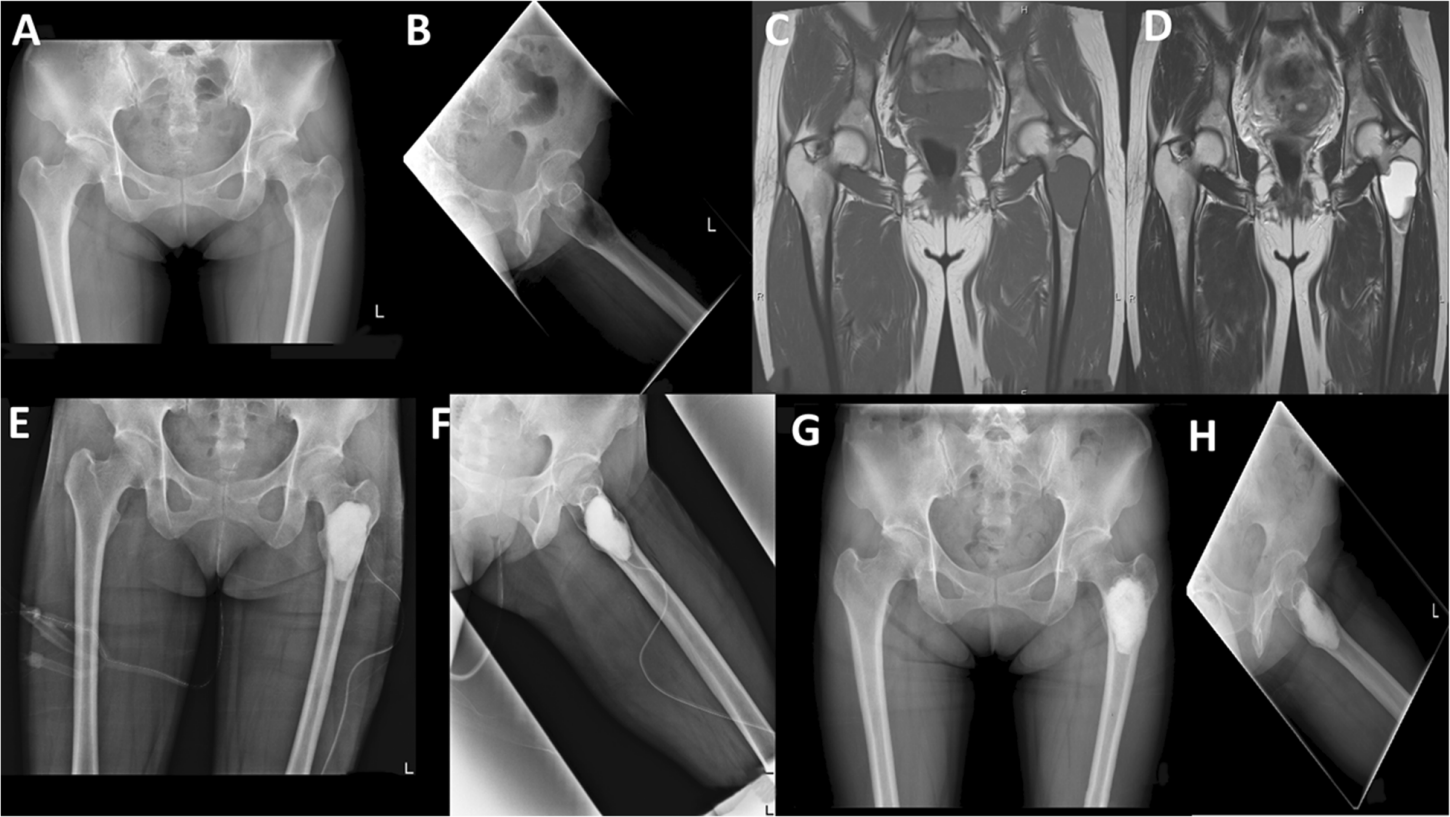
Case presentation representing a 37-year-old female with fibrous dysplasia of the left proximal femur. **a**, **b** Anteroposterior and lateral radiographs showing fibrous dysplasia of left proximal femur. **c**, **d** MRI of proximal femur showing the fibrous dysplasia lesion with marked cystic change. **e**, **f** Post-operative radiographs showing the lesion after curettage and implantation of alpha tricalcium phosphate. **g**, **h** Radiographs taken at final follow-up (4 years after surgery)

Through the cortical window, adequate curettage of the lesion was performed. A high-speed burr may be used to perform an extended curettage in cases of aggressive benign lesions such as GCT and chondroblastoma with the use of phenol and ethanol as local adjuvants to reduce local recurrence rate. After complete curettage, implantation of the synthetic bone graft within the bone defect was performed (Fig. 1). If internal fixation was indicated, we apply first the synthetic bone graft and then the internal fixation was performed.

Range of motion exercises for patients were started 24 h after their operation and partial weight-bearing with crutches was allowed 24 h to 1 week after surgery. Patients were followed-up with serial radiographs at approximately 6 weeks, every 3 months for 2 years, every 6 months for another 3 years, and then once each year. Progressive weight-bearing was allowed from 1 week to 12 weeks depending on the initial size and location of the lesion, if internal fixation was performed or not and radiographic consolidation of the bone defect.

## Results

The mean operative time was 143 min (range, 80–245 min). At the last follow-up, all patients had regained normal unrestricted activity without pain at the operation site. Patients treated with β-TCP achieved radiographic consolidation of the bone defects within 1 year after the surgery and those treated with α-TCP or HA experienced no progression nor recurrence of the lesions.

Local tumor recurrence was observed in one patient with GCT 5 years after the surgery. The lesion was treated with recurettage and implantation of α-TCP within the bone defect, and the patient regained full physical function by final follow-up at 3 years after second surgery. Post-operative pathological fracture was occurred in one patient with SBC of the subtrochanteric region 1 month after surgery and, the patient was treated with open reduction and internal fixation using a locked plate and screws. No post-operative infection was observed.

## Discussion

In our study, we reported the outcomes for the patients with benign lytic lesions of the proximal femur who underwent surgical treatment by curettage followed by implantation of a synthetic bone graft. We observed only one patient with GCT that developed local recurrence 5 years after surgery and he was treated successfully with recurettage followed by implantation of α-TCP. At the last follow-up, all patients had achieved full physical function.

There is controversy about the ideal material used for reconstruction of the bone defect after curettage of the benign lesion of the proximal femur. Implantation of bone defects with synthetic bone substitute has many advantages; it is a relatively simple technique, provides an unlimited source of bone graft, acts as a scaffold for new bone formation, and avoids the transmission of infections and complications at the donor site.

Autologous non-vascularized fibular grafts were recommended by some authors in the management of benign lesions of the proximal femur [2, 8]. Although autologous fibular graft is a good bone substitute, it has the disadvantage of donor site complications such as bleeding, nerve palsy, and stress fracture. Also, it is not always available and comes with high risk of tumor implantation into the donor site [7]. Further, revascularization by creeping substitution causes reduction in the structural strength of the fibular graft in the first weeks after surgery, which entails the restriction of patients’ activities and weight-bearing for a long time [2].

Vascularized autologous fibular graft is a superior graft to its neovascularized counterpart as it does not undergo resorption and it can remodel in a similar fashion to normal bone [11, 12]. However, the use of vascularized grafts has the disadvantages of a more complex procedure, prolonged operative time, and the need for microsurgical anastomosis [2].

Autologous cancellous bone graft has the advantages of rapid incorporation, easy revascularization and not immunogenic [2], but it does not provide adequate postoperative structural support, and if it is used alone, it has a high failure rate up to 48%. Furthermore, it has limited supply for large bony defects [4]. So that this type of graft was usually used in conjunction with autologous or allogenic strut grafts and internal fixation to overcome these shortages [1, 5].

Allografts may be used for reconstruction of bony defects after curettage as they have the advantage of no donor site complications, but they have the risk of transmission of infectious disease [13–15], a reduced rate of incorporation [2], and requires availability of a bone bank system which may not be available in some areas of the world due to socio-religious reasons.

In most cases, for reconstruction of bone defects after curettage, we used either α-TCP or β-TCP depending on the size of the lesion and age of the patient. We preferred to use β-TCP for small lesions and in pediatric patients as it acts as a scaffold for new bone formation with a gradual increase in mechanical strength of the reconstructed bone defect. α-TCP or calcium phosphate cement (CPC) was used for larger, recurrent or aggressive lesions for rapid restoration of mechanical strength.

Internal fixation was indicated only in three cases with large lesions that presented with preoperative impending pathological fracture due to significant endosteal erosion of the cortex and marked thinning of the cortical wall. We reported one patient with post-operative pathological fracture who was treated successfully with open reduction and internal fixation using a locked plate and screws. Some previous studies recommended reinforcement of the proximal femur with internal fixation to prevent postoperative fracture in the management of benign bone tumors that affects more than 50% of the diameter of the femoral neck [6, 7]. But overindication of internal fixation has disadvantages such as prolonged operative time, more operative bleeding, more tissue injury, higher infection rates, tissue irritation with chronic pain, and in pediatric patients, another operation may be needed to remove the osteosynthesis [5]. On the other hand, inadequate fixation my result in increased risk of postoperative fracture so the addition of internal fixation should be carefully planned.

Nakamura et al. [7] reported the outcomes of 13 patients with benign bone tumors treated with synthetic bone graft and compression hip screws. They reported that average blood loss was 1088 mL, and blood transfusion was required in eight cases. The mean operative time was 167 min. They reported post-operative superficial wound infection in one patient and chronic hip pain in another patient due to soft tissue irritation which was managed by removal of the dynamic hip screw 7 years after the index surgery.

In our study, the choice of surgical approach depended on the location of the lesion within the proximal femur. If the lesion involved the trochanteric or subtrochanteric region, the operation was performed through a lateral approach that provided direct access to the lesion, but if the lesion involved only the neck or head of the femur, the operation was performed through an anterior approach as this approach allowed direct access to the tumor with adequate exposure of the femoral head and neck without dislocation of the hip and complete curettage of the lesion [16]. However, this approach should be performed with caution to avoid injury of lateral circumflex femoral artery and tumor implantation around the femoral vessels [7]. Therefore, the approach should be carefully planned to avoid these complications.

In our study, there are eight cases of fibrous dysplasia of the proximal femur.

They are of monostotic type. Fibrous dysplasia is a benign intramedullary fibro-osseous lesion that occurs because of failure of maturation of primitive bone into lamellar bone resulting into a lesion of immature trabeculae that imbedded in dysplastic fibrous tissue [17]. Fibrous dysplasia can occur as a monostotic or polyostotic form. Monostotic lesions are active only until skeletal maturity is achieved [18]. We treated symptomatic cases of monostotic fibrous dysplasia of proximal femur by curettage followed by implantation of alpha tricalcium phosphate to control the lesions until skeletal maturity and normalization is reached. These cases showed no progression of the lesion at final follow-up.

This study has some limitations. It is a retrospective level IV evidence study with limited number of patients. So, a larger study may be needed in the future.

## Conclusion

We concluded that the treatment of benign lytic lesions of the proximal femur either primary or recurrent using synthetic bone graft is a safe and effective method, and the addition of internal fixation should be carefully planned.

## Abbreviations

CPC: Calcium phosphate cement
CT: Computed tomography
GCT: Giant cell tumor
HA: Hydroxyapatite
MRI: Magnetic resonance imaging
SBC: Simple bone cyst
α-TCP: Alpha tricalcium phosphate
β-TCP: Beta tricalcium phosphate
